# Evaluating the Knowledge of Home Caregivers on Pressure Ulcer Signs and Prevention in Elderly Patients in Al Ahsa

**DOI:** 10.7759/cureus.73199

**Published:** 2024-11-07

**Authors:** Abdulhadi A Alabdulhadi, Maryam Albrahim, Fatemah A Albshr, Ali A Al jaber, Fatemah A Al Sahaf, Eman A Aldrees, Ruqayyah M Althani, Thikra Alibrahem, Aminah N Alismail

**Affiliations:** 1 Home Health Care, Prince Saud Bin Jalawi Hospital - Al Ahsa Health Cluster, Al Ahsa, SAU; 2 Home Health Care, Prince Saud Bin Jalawi Hospital -Al Ahsa Health Cluster, Al Ahsa, SAU; 3 Delivery Room, King Faisal Hospital - Al Ahsa Health Cluster, Al Ahsa, SAU

**Keywords:** al ahsa, elderly care, home caregivers, knowledge assessment, pressure ulcer prevention

## Abstract

Background

Pressure ulcers are significant health issues affecting elderly patients, leading to severe complications, prolonged hospital stays, and high healthcare costs. Preventive care by home caregivers plays a crucial role in mitigating the development of pressure ulcers.

Objective

This study aims to assess home caregivers' knowledge of pressure ulcer prevention and investigate the factors associated with knowledge levels in elderly patients in Al Ahsa, Saudi Arabia.

Methods

A cross-sectional study was conducted involving 231 home caregivers of elderly patients registered in Home Health Care, Al-Ahsa Health Cluster. Data were collected using an online questionnaire assessing demographic information and knowledge of pressure ulcer prevention, adapted from the Pressure Ulcer Knowledge Assessment Tool (PUKAT) 2.0. Data analysis was performed using SPSS version 29.0 (IBM Corp., Armonk, USA), with a Chi-square test used for bivariate analysis of categorical outcomes.

Results

The majority of caregivers were female (64.5%), aged between 35 and 50 years (47.6%), and primarily family members (83.5%). Most caregivers had been providing care for 5 years or less (79.7%), with 67.5% never having received training in pressure ulcer prevention. Knowledge levels were generally sufficient, with 87.0% of caregivers showing adequate knowledge of pressure ulcer prevention. Significant associations were found between knowledge levels and caregiving duration (p=0.006) as well as training in pressure ulcer prevention (p=0.001). However, other demographic factors, such as age and gender, were not significantly associated with knowledge levels.

Conclusion

The study found that most home caregivers possess sufficient knowledge of pressure ulcer prevention, although training and caregiving duration significantly influence knowledge levels. These findings underscore the need for comprehensive training programs to improve caregivers' knowledge and practice, thereby enhancing the quality of care for elderly patients and reducing the incidence of pressure ulcers.

## Introduction

Pressure ulcers, pressure sores, decubitus ulcers, or bed sores, are localized injuries that affect the skin and underlying tissues, that are caused by prolonged pressure, friction, or shear forces, leading to tissue ischemia and necrosis [1,2]. Such injuries from pressure ulcers range from mild redness of the skin to severe wounds with exposed muscles or bones [2]. Multiple healthcare settings have reported pressure ulcers of varying incidence among patients of different age groups [3].

The world population over 65 years of age was estimated at 8.5% in 2015 and is predicted to rise to 16.7% by 2050 [4]. This increase in the number of elderly individuals has a myriad of implications, one of which is an increase in the prevalence of conditions like Pressure ulcers. International pressure ulcer prevalence surveys conducted between 1989 and 2005 indicated an overall prevalence of approximately 15% in healthcare facilities [5]. Another study showed that approximately two-thirds of pressure ulcers are reported in elderly individuals aged between 60 and 80 years [6].

Bed sores in elderly patients have been reported to result in poor quality of life, pain, extended hospital stays, high healthcare costs, poor rehabilitation outcomes, and potentially serious complications [7]. Pressure ulcers are preventable, however, they remain a major issue for elderly patients in various healthcare settings, especially among the chronically ill [8]. Low serum albumin levels, malnutrition, impaired mobility, and cognitive changes are some of the risk factors contributing to the development of pressure ulcers in the elderly population [9].

Preventing pressure ulcers is pivotal due to their significant morbidity and association with high mortality rates [10]. The preventive practices involve various strategies such as the use of alternating pressure air surfaces, conservative treatment methods, and the optimization of support interfaces [1,10]. Using risk assessment scales, educational programs, and technology-based interventions have also been reported to help in identifying individuals at risk and improving preventive measures [11].

Home care for the elderly involves a diverse group of individuals who play a crucial role in preventing pressure ulcers in this population. These caregivers include formal care workers in long-term care facilities, family members, and paid home-care givers. In long-term care facilities, registered nurses educate care workers to actively participate in pressure ulcer prevention activities because of the high ratio of elderly persons to care workers [12]. In the home care setting, family members are typically the primary care providers for the elderly [13]. They are involved in various aspects of care, including monitoring vital signs, and medication management [14].

Prevention of pressure ulcers is crucial in the care of the elderly. These ulcers can be a major predictor of mortality in elderly patients living at home or in nursing homes [15]. Family members and formal care providers are essential in preventing pressure ulcers through knowledge and practice on factors like pressure-relieving devices, position changes, skin lubrication, and ensuring adequate nutrition for the patient [16]. Proper preventive nursing care is crucial for elderly individuals at risk of pressure ulcers. However, family members often find it challenging to manage elderly patients with pressure ulcers at home [17,18].

The association between home caregivers' knowledge of pressure ulcer prevention and the development of pressure ulcers in elderly patients is a crucial aspect of care. Research has demonstrated that caregivers' knowledge and attitudes towards pressure ulcer prevention significantly influence the occurrence and management of pressure ulcers in the elderly [19,20]. A study by Dille and Mengistu, 2015 showed that nurses' knowledge and attitudes are essential factors for the prevention of pressure ulcer formation [20]. Furthermore, multiple other studies have reported insufficient knowledge about pressure ulcer prevention among caregivers, including nurses and nursing assistants [19]. Meanwhile, interventions that enhance caregivers' knowledge and practice of pressure ulcer prevention have proven effective in reducing the incidence of pressure ulcers [21].

This study aims to assess home caregivers' knowledge of pressure ulcer prevention and investigate the factors associated with knowledge levels. Understanding these factors could lead to better education and training for caregivers, potentially reducing pressure ulcers, improving elderly patients' quality of life, and lowering healthcare costs.

## Materials and methods

Study design and area

This study employed a cross-sectional design and was conducted in Al Ahsa, a region in eastern Saudi Arabia. The focus was on home caregivers who work with elderly patients registered in Home Health Care, Al Ahsa Health Cluster.

Study population

The study population consisted of home caregivers of elderly patients registered in Home Health Care, Al Ahsa Health Cluster. The initial sample size estimation was 212, calculated using an online sample size calculator (Raosoft.com), considering the total number of home caregivers (470), with a 5% margin of error, 95% confidence level, and 50% response distribution. To enhance generalizability and account for potential missing data, the sample size was increased to a final sample size of 231 participants.

The eligibility criteria for the study included home caregivers aged 18 years or older providing care to elderly patients in Al Ahsa, Saudi Arabia, regardless of gender, and those willing to participate in the study. Home caregivers under 18 years old and individuals unwilling to participate were excluded from the study.

Sampling technique

A purposive sampling technique was utilized due to the specific nature of the population, i.e., home caregivers of elderly patients. This method allowed for the selection of individuals who met the inclusion criteria and were able to provide valuable information due to their direct involvement in elderly care. Recruitment involved contacting potential participants through the Home Health Care database affiliated with Al-Ahsa Health Cluster.

Data collection

Data were collected through an online questionnaire, which included two sections: demographic information and knowledge of pressure ulcer prevention. The demographic section gathered information about the caregivers' age, gender, relationship to the elderly individual they cared for, and any formal training or education they had received in elderly care or pressure ulcer prevention.

The second section, adapted from the validated Pressure Ulcer Knowledge Assessment Tool (PUKAT) 2.0, assessed the caregivers' understanding of pressure ulcers and their prevention [22]. Questions covered causes, risk factors, common locations on the body, prevention strategies, recognition of early signs, and the role of nutrition. Each question had multiple-choice answers, with one correct answer earning one point. Scores ranged from 0 to 12, with scores above 7 (more than 60%) considered sufficient knowledge in pressure ulcer prevention.

Data analysis

Data were analyzed using the Statistical Package for the Social Sciences version 29 for MacOS (IBM Corp., Armonk, NY). Proportions and frequencies summarized categorical variables. The Chi-square test was used for bivariate analysis of categorical outcomes. A p-value less than 0.05 was considered significant, and a 95% confidence level was used for inferences.

Ethical considerations

Ethical approval was obtained from the Institutional Review Board (IRB) at Al-Ahsa Health Cluster (H-05-HS-135) before data collection commenced. Participants were provided with a statement outlining the study's nature and purpose to obtain their consent before completing the questionnaire. Data were handled with utmost confidentiality, stored securely, and used exclusively for research purposes.

## Results

Demographic characteristics

The demographic characteristics of the included study participants (n=231) are presented in Table 1. The majority of caregivers were aged between 35 and 50 years (n=110, 47.6%), followed by those aged under 35 years (n=93, 40.3%), and a smaller proportion over 50 years (n=28, 12.1%). In terms of gender, 64.5% of the participants were female (n=149), whereas 35.5% were male (n=82). The relationship of caregivers to the elderly patients showed that the majority were family members (n=193, 83.5%), followed by other categories (n=23, 10.0%), and paid caregivers (n=15, 6.5%).

**Table 1:** : Demographic characteristics Demographic profile of home caregivers. The data has been presented as frequencies and proportions.

n=231		N	%
Age (Year)	<35	93	40.3%
	35 - 50	110	47.6%
	>50	28	12.1%
Gender	Male	82	35.5%
	Female	149	64.5%
Relationship	Family member	193	83.5%
	Paid caregiver	15	6.5%
	Other	23	10.0%

Caregiving experience and training

Most caregivers had been providing care for 5 years or less (n=184, 79.7%), while 15.2% had been caregiving for 6 to 10 years (n=35), and 5.1% for over 10 years (n=12). A significant portion of caregivers (67.5%) had never received training in pressure ulcer prevention (n=156), whereas 32.5% had received such training (n=75) (Table 2).

**Table 2:** Caregiving experience and training Caregiving Experience, Training, and Knowledge Levels of Home Caregivers. The data has been presented as frequencies and proportions.

n=231		N	%
Caregiving duration (Year)	≤5	184	79.7%
	6 - 10	35	15.2%
	>10	12	5.1%
Had training in pressure ulcer prevention	No	156	67.5%
	Yes	75	32.5%
Knowledge level	Insufficient	30	13.0%
	Sufficient	201	87.0%
Understating of pressure ulcers	Insufficient	41	11.3%
	Sufficient	190	88.7%
Knowledge of pressure ulcer signs	Insufficient	76	13.4%
	Sufficient	155	86.6%
Knowledge of pressure ulcer prevention	Insufficient	28	10.4%
	Sufficient	203	89.6%

Regarding overall knowledge levels, 87.0% of caregivers had sufficient knowledge (n=201), and 13.0% had insufficient knowledge (n=30). Understanding of pressure ulcers was sufficient in 88.7% of the participants (n=190), compared to 11.3% with insufficient understanding (n=41). When it came to knowledge of pressure ulcer signs, 86.6% of caregivers had sufficient knowledge (n=155), while 13.4% had insufficient knowledge (n=76). For pressure ulcer prevention knowledge, 89.6% of caregivers were sufficient (n=203), and 10.4% were insufficient (n=28) (Table 2).

Factors associated with knowledge level

Table 3 highlights the factors associated with the knowledge level of caregivers. Age did not show a significant association with knowledge level (*p*=0.358). Gender also was not significantly associated with knowledge levels (*p*=0.156). However, the relationship of the caregiver to the patient approached significance (*p*=0.056), with family members more likely to have sufficient knowledge compared to paid caregivers and others (Table 3).

**Table 3:** Factors associated with knowledge level X^2^: Chi-Square test. *. Association is significant at* p*-value <0.05. The relationship between demographic and caregiving factors and the knowledge level of home caregivers, based on scores from the Pressure Ulcer Knowledge Assessment Tool (PUKAT) 2.0 [22]. Scores above 60% indicate sufficient knowledge. The data has been presented as frequencies and proportions.

n=231		Knowledge level					
		Insufficient		Sufficient			
		N	%	N	%	p-value	X^2^ value
Age (Year)	<35	11	36.7%	82	40.8%	0.358	2.059
	35 – 50	13	43.3%	97	48.3%		
	>50	6	20.0%	22	10.9%		
Gender	Male	7	23.3%	75	37.3%	0.156	2.228
	Female	23	76.7%	126	62.7%		
Relationship	Family member	22	73.3%	171	85.0%	0.056	5.211
	Paid caregiver	5	16.7%	10	5.0%		
	Other	3	10.0%	20	10.0%		
Caregiving duration (Year)	≤5	24	80.0%	160	79.6%	0.006*	10.092
	6 - 10	1	3.3%	34	16.9%		
	>10	5	16.7%	7	3.5%		
Had training in pressure ulcer prevention	No	28	93.3%	128	63.7%	0.001*	10.468
	Yes	2	6.7%	73	36.3%		

Caregiving duration showed a significant association (*p*=0.006), with those caregiving for 5 years or less tending to have sufficient knowledge (n=160, 79.6%). Caregivers with 6 to 10 years of experience and over 10 years of experience showed varied levels of sufficient knowledge (n=34, 16.9%, and n=7, 3.5%, respectively) (Table 3).

Training in pressure ulcer prevention was significantly associated with knowledge levels (*p*=0.001). Caregivers who had received training were more likely to have sufficient knowledge (n=73, 36.3%) compared to those who had not received training (Table 3).

## Discussion

Pressure ulcers (PU) also known as decubitus ulcers affect approximately 3 million people in the United States each year [23]. Effective prevention and management of pressure ulcers are critical for improving patient's quality of life. Our study investigated the association between home caregivers' knowledge of PU prevention and the incidence of PUs in elderly patients at home.

A systematic review of eight studies included 927 caregivers with a mean age of 40.5 years (61.87% women), who reported an average knowledge of 53.7% regarding the prevention of pressure ulcers [24]. In contrast, in our study which included predominantly middle-aged participants between 35 to 50 years with 64.5% females; 87% of participants had sufficient knowledge. These findings highlight the significant variability in caregiver’s knowledge across different studies. For instance, Mohammed et al. reported that 29% of caregivers had satisfactory knowledge compared to 71% who did not have good knowledge [25]. Another study focusing on caregivers of immobilized patients reported that 68% had average knowledge, 24% had good knowledge, and 8% of them had poor knowledge of pressure ulcers [26]. Farzan et al. studied risk factors affecting the knowledge of caregivers regarding the prevention of pressure ulcers, there was a negative relationship with age suggesting that younger patients have more knowledge. Various demographic factors, including marital status, gender, occupation, and level of education showed a significant correlation with caregiver knowledge of pressure ulcers [24]. The training programs have a crucial role in the improvement of knowledge related to the prevention of pressure ulcers among caregivers [27]. Although a significant number (67%) of our participants had never received formal training, a majority of caregivers still have sufficient knowledge and awareness about pressure ulcers. It is essential to prioritize the prevention of pressure ulcers when caring for patients with prolonged immobility, such as those with fragility fractures [28]. Therefore, it is important to train caregivers, especially in elderly settings which eventually contributes to preventing pressure ulcers. A study by Antony et al. revealed that 50% of participants had poor knowledge, 40% had average knowledge, and only 10% had good knowledge on the prevention of pressure ulcers [29]. In contrast, 89% of caregivers in our case had sufficient knowledge to prevent pressure ulcers. 

There are certain risk factors in the development of pressure ulcers including limited mobility, poor health and circulation, poor nutrition, unmanaged incontinence, impairment of thinking, nerve damage that reduces feeling pain or pressure, and impairment of thinking and reasoning [30]. Although the previous studies have not been analyzed, our study identified the personal relationship of the caregiver with the patient had improved caregiving, and better knowledge to prevent pressure ulcers in comparison with paid caregivers. Farzan et al. also elaborated on the type of relationship that has a significant role in the knowledge of pressure ulcer prevention [24]. Caregivers, whether family members or paid professionals have a crucial role in the prevention of bedsores in bed-bound patients. Patients and their caregivers should receive education on managing their condition in hospital and their homes. They should also have familiarity with warning signs including skin discoloration, ulceration, foul smell from the ulcers, and numbness of body areas [31]. The vast majority (86%) of our patients are sufficiently aware of signs of pressure ulcers. Access to preventive tools such as specific types of beds, overlays, and mattresses has a significant role in caregiving at a professional level. The inadequacy of these preventive measures negatively affects the prevention and treatment of pressure ulcers [32].

Real-time monitoring of pressure is crucial in the prevention of pressure ulcers. The continuous monitoring of the development and progression of PU also has a significant role. However, it increases the cost and time leading to the solution of developing a sensor-based system that minimizes the requirement of healthcare professionals [33]. In combination, lack of knowledge, inadequate preventive measure access, and inconsistent monitoring by caregivers significantly challenge the prevention and management of pressure ulcers.

Recommendations

Based on the findings of this study, it is recommended that comprehensive training programs on pressure ulcer prevention be developed and implemented for home caregivers. These programs should include practical sessions and continuous education to ensure that caregivers remain updated on the best practices. Additionally, healthcare facilities should provide caregivers with access to preventive tools such as specialized mattresses and pressure-relieving devices. Regular assessments of caregivers' knowledge and skills should be conducted to identify areas for improvement. Finally, fostering collaboration between healthcare professionals and caregivers can enhance the overall quality of care and further reduce the incidence of pressure ulcers in elderly patients.

Strengths

This study has several notable strengths. Firstly, the study utilized a validated assessment tool (PUKAT 2.0) to measure caregivers' knowledge of pressure ulcer prevention, ensuring the accuracy of the data collected. Secondly, the research provides valuable insights into the factors associated with caregivers' knowledge levels, such as caregiving duration and training in pressure ulcer prevention. These findings can inform targeted interventions to improve caregivers' knowledge and skills. Lastly, the study's focus on home caregivers in Al Ahsa, Saudi Arabia, contributes to the limited body of research on pressure ulcer prevention in this specific context, potentially informing local healthcare policies and practices.

Limitations

This study has several limitations that should be acknowledged. Firstly, the cross-sectional design restricts the ability to establish causality between caregivers' knowledge and the development of pressure ulcers. Moreover, the study did not account for other factors such as socioeconomic status, cultural differences, or variations in healthcare access, which might also influence the outcomes. Future research should consider longitudinal designs, larger and more diverse samples, and objective measures to validate and expand upon these findings.

## Conclusions

In conclusion, this study emphasizes the crucial role of home caregivers' knowledge in preventing pressure ulcers among elderly patients. It was found that the majority of caregivers have sufficient knowledge of pressure ulcers, including their signs and preventive measures. Key factors such as caregiving duration and training in pressure ulcer prevention were associated with knowledge levels. These findings underscore the importance of targeted educational programs and continuous training to equip caregivers with the necessary skills and knowledge to effectively prevent pressure ulcers, thereby improving the quality of life for elderly patients and reducing healthcare costs.
